# The Use of Local Medial Plantar Artery Flap for Heel Reconstruction: A Systematic Review

**DOI:** 10.7759/cureus.9880

**Published:** 2020-08-19

**Authors:** Jude L Opoku-Agyeman, Amber Allen, Kayla Humenansky

**Affiliations:** 1 Plastic Surgery, Philadelphia College of Osteopathic Medicine, Philadelphia, USA; 2 Foot and Ankle Surgery, Private practice, Philadelphia, USA; 3 Plastic and Reconstructive Surgery, Philadelphia College of Osteopathic Medicine, Philadelphia, USA

**Keywords:** heel reconstruction, instep flap, medial plantar artery flap, heel wounds

## Abstract

Background: Soft tissue reconstruction of the heel represents a daunting challenge for reconstructive surgeons, given the weight-bearing role and anatomical properties of the glabrous skin on the plantar surface. For soft tissue defects in this area, the medial plantar artery (MPA) flap has been described as an optimal reconstructive option. Many studies have reported on the use of the medial plantar artery flap for soft tissue coverage of the heel. There currently exists no systematic review on the topic.

Aim: The aim of this article is to review the literature on the use of local medial plantar artery flap for heel reconstruction with a focus on overall flap viability and selected outcomes.

Method: The authors performed a systematic literature review using EMBASE, Cochrane Library, Ovid Medicine, MEDLINE, Google Scholar, PubMed database, and grey literature. Studies were identified between 1981 and 2019. Peer-reviewed articles published in the English language were included. Articles were eligible if they contained original clinical outcomes on patients who underwent local medial plantar artery flap for reconstruction of heel defects.

Results: A total of 135 unique studies were identified. Eighteen (18) articles were included in the review and analyses, yielding a total of 277 local medial plantar artery flaps for heel coverage. The most common etiology for the reconstructed heel defect was ulcers (45.3%) followed by trauma (35.8%). The overall complete flap survival rate was n=272/277 (98.2%). The incidence of minor flap complication was n=26/277 (9.4%). Most of the flaps maintained protective sensation (n=147/148 [99.3%]), although the protective sensation tended to be inferior to the contralateral normal side. The rate of donor site morbidity was n=14/269 (5.2%).

Conclusion: Local medial plantar artery flap for heel defect reconstruction is associated with a very high flap survival rate with very few flap related complications including donor site complications.

## Introduction and background

Heel soft tissue defects present a challenge for reconstructive surgeons. This challenge has been attributed to the poor availability of locoregional tissue to perform the reconstruction [1]. The plantar foot, including the heel, have unique intrinsic properties to accommodate the high compressive load and shearing forces exerted during standing and ambulation [2]. In addition to a large fat pad, the heel has thicker skin compared to the non-weight bearing surfaces of the plantar foot, allowing it to withstand more pressure and force [3]. It is therefore important and equally challenging to reconstruct the plantar foot while maintaining function after surgery [4]. Additionally, the plantar foot has minimal skin laxity thus making primary closure nearly impossible. This leads to the utilization of other options on the reconstruction ladder [3]. Normal ambulation is dependent on many factors, including ample and durable soft tissue coverage, as well as protective sensation. Ideally, reconstruction of heel defects involves reconstruction of “like with like” tissue. The ideal option for reconstruction should be durable, sensate, and associated with low morbidity [5].

Reconstruction of heel defects can be achieved with many options including skin grafts, local random flaps, regional flaps, cross-leg flaps, and free tissue transfer. The use of skin grafts is simple but does not provide durable tissue for the weight-bearing surface of the heel. Additionally, skin grafts are insensate. Consequently, the use of skin grafts has been limited to providing coverage for adipofascial or free-muscle flaps [1]. Transposition flaps elevated superficial to the plantar fascia require “delay” due to their unreliable random pattern blood supply. Plantar transposition flaps are based on perforating vessels from the medial and lateral plantar arteries and veins [6]. Free tissue transfer has been used for heel reconstruction [7]. It is a viable option for cases where there are no local or regional flaps available. Free tissue transfer is associated with more potential complications including donor site morbidity. Additionally, free tissue transfer is technically challenging and requires expertise in microsurgery.

The use of ipsilateral medial plantar artery flaps (MPA flaps) has gained a lot of interest in the past and present. Many options exist for foot reconstruction; however, for heel reconstruction, the two most commonly utilized reconstructive options are the medial plantar artery flap and the reverse sural artery flap. The medial plantar artery island flap was described by Harrison and Morgan in 1981 [6]. The medial plantar artery flap or instep flap involves harvesting tissue from the instep of the foot on a vascular pedicle or a perforator. Its neurovascular supply is based on the medial plantar artery and cutaneous digital branches of the medial plantar nerve [8]. It can be raised as a fasciocutaneous or musculocutaneous flap.

The MPA flap provides a composite of tissue very similar to that of the plantar heel, with a relatively expendable non-weight bearing donor site [9]. It has facilitated heel coverage since its development [10]. It results in thick, weight-bearing, sensible skin, resistant to friction [6]. For the purpose of this study, the heel consists of the weight-bearing heel (anterior heel), and the non-weight bearing heel, made of the calcaneo-tendinous insertion of the Achilles tendon.

In this systematic review, we examine the current literature on the use of ipsilateral local MPA flaps for reconstruction of heel defects, focusing on the rate of complete flap survival and minor flap complications. To the best of our knowledge, this will be the first systematic review on this topic.

## Review

Methods

Research Design

**Figure 1:**
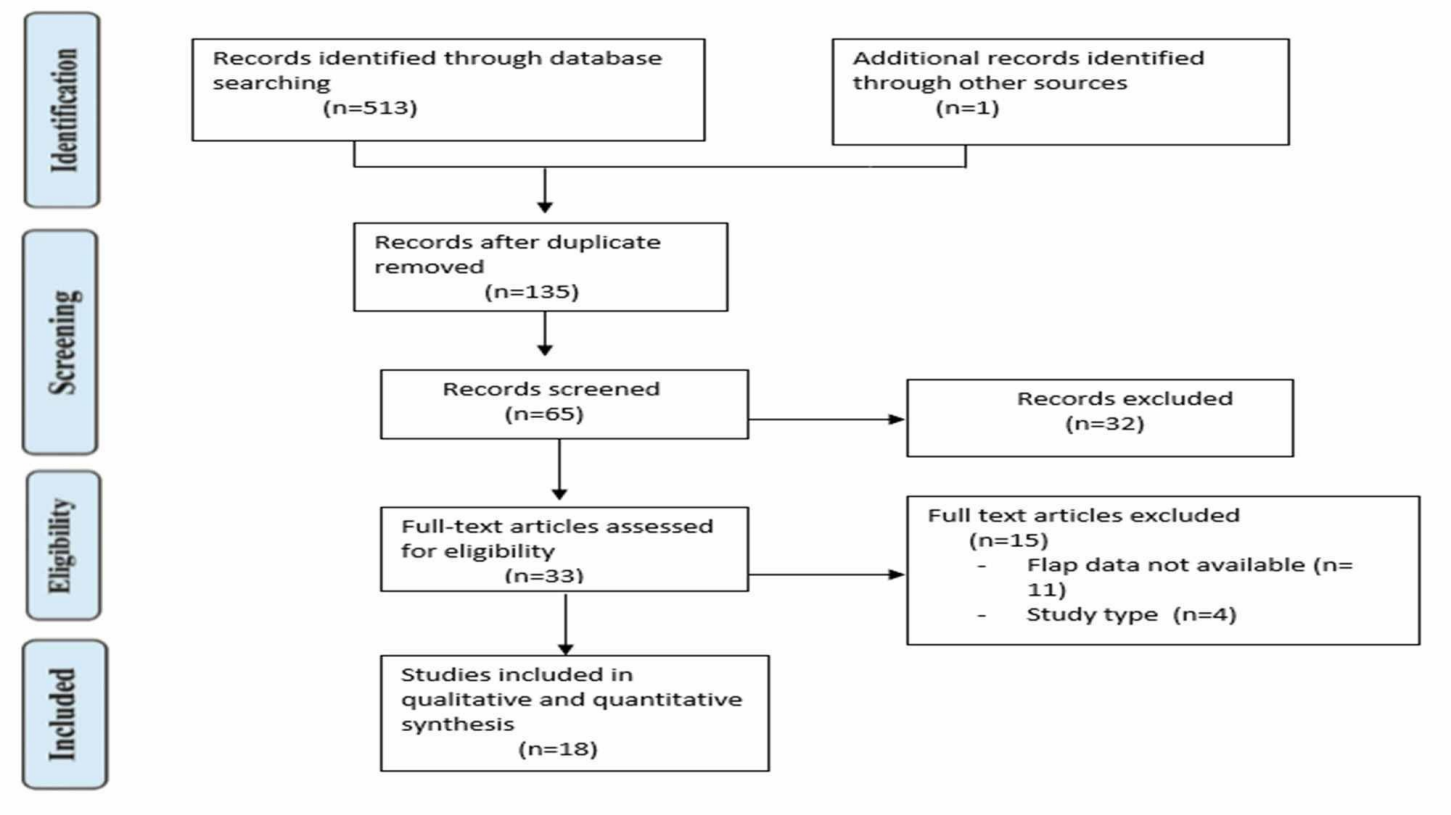
PRISMA flow chart PRISMA, Preferred Reporting Items for Systematic Review and Meta-Analyses

Our review followed guidelines published by the Cochrane Collaboration and the Preferred Reporting Items for Systematic Reviews and Meta-Analyses criteria (PRISMA) [11,12]. This study complies with the principles of the Declaration of Helsinki. There is no protocol for this review. The PRISMA flow chart is shown in Figure 1. 

Search Methodology and Strategy

The following electronic databases were searched: EMBASE, Cochrane Library, Ovid Medicine, MEDLINE, Google Scholar, and PubMed. We identified articles in the grey literature on the topic by doing searches on European Union Clinical Trials Register, Open trials, Prospero, Opengrey, Clinical Trial.gov, ProQuest, and open thesis.

The search strategy was developed to locate articles related specifically to the use of local medial plantar artery flap for heel reconstruction. The search used the English language keywords combined with Boolean logical operators. The following terms were used without any limits: “medial plantar artery flap” or “instep flap” or “foot flap” and “heel reconstruction” or “foot wound” or “heel wound” or “foot reconstruction.” If the full text or abstract of a reference was not found, authors of eligible studies were contacted for full text or exclusion was based on available data. Reference lists of reviews, editorials and commentaries, and case reports, as well as included articles, were screened for relevant publications.

Selection Criteria

The inclusion and exclusion criteria were defined before data collection was carried out. Studies that evaluated the use of local MPA flap in heel reconstruction were included. All studies must have clearly stated that the patient underwent MPA flap for heel reconstruction; if this was unclear, the article was excluded. Case series with more than four cases, retrospective, and prospective studies were included. Studies were excluded if they were not published in the English language, did not clearly state the number of MPA flaps, did not report the appropriate outcomes as stated, or were reviews or commentaries. Studies that reported outcomes on free MPA flaps and cross leg MPA flaps were excluded. Studies were limited to peer reviewed studies published after 1981, when the MPA flap was first described.

Data Extraction and Synthesis

Data from each included study was collected by two authors independently to a specifically developed data extraction form using Microsoft Excel 2010. We predefined a minimum set of information that must be extractable from the publication: flap survival rate, minor flap complications and number of medial plantar artery flaps. If the minimum dataset was not provided, corresponding authors were contacted to obtain the missing data in order to appropriately describe the study results. If information on missing data could not be obtained, the article was excluded.

Information was collected on study characteristics (author, year of publication, study design, sample size, duration of follow-up) and participant characteristics (age, numbers of flaps, largest dimension of heel defect). Outcomes data (flap survival rate, minor flap complications, loss of protective sensation, donor site complications, and ambulatory status at the time of follow up) were also extracted.

We planned to perform a qualitative synthesis of the findings, outlining the characteristics of included studies, indications for flap use, and summarized data of outcomes. Since this study was specifically looking at outcomes of heel reconstruction with MPA flap, independent of any risk factors, we did not intend to perform a meta-analysis. However, a pooled analysis of primary and secondary endpoints was planned, to determine the rate of incidence of complications within our study population. This was done by entering the pooled data in Microsoft Excel (2010). The rate of complete flap survival and other outcomes was then determined.

Assessment of Study Quality and Bias in Included Studies

The methodological quality of studies was assessed using the Grading of Recommendation Assessment, Development and Evaluate (GRADE) system [13] and the American Society of Plastic Surgery (ASPS) level of evidence scale [14].

Results

Our search in the various databases yielded 514 publications. One hundred thirty-five cases remained after removal of duplicates. The characteristics of includes studies [10,15-31], and the results of quality analysis of each study are illustrated in Table 1. Of the 18 articles included in the qualitative and pooled analysis, 16 of 18 were level 4 studies [10,15-19,21-28,30,31] and two of 10 were level 3 studies [20,2]. There were no randomized controlled clinical studies. All studies were published between 1984 and 2018. The GRADE scores ranged from very low to moderate.

**Table 1:** Characteristics of included studies ASPS, American Society of Plastic Surgeons GRADE, Grading of Recommendation Assessment, Development and Evaluation

Publication	Year of publication	Type of study	ASPS Level of evidence	GRADE quality of evidence
Reading [15]	1984	Case series	4	Very low
Amarante et al. [16]	1986	Case series	4	Very low
Miyamoto [17]	1987	Prospective review	4	Low
Baker et al. [18]	1990	Retrospective review	4	Low
Gravem [19]	1991	Retrospective Review	4	Low
Rashid et al. [20]	2003	Prospective comparative	3	Moderate
Benito-Ruiz et al. [21]	2004	Retrospective review	4	Low
Mourougayan [22]	2006	Retrospective review	4	Low
Schwarz et al. [10]	2006	Prospective	4	Moderate
Chaudhry et al. [23]	2008	Prospective study	4	Low
Oh et al. [24]	2011	Retrospective review	4	Low
Yang et al. [25]	2011	Retrospective review	4	Low
Siddiqi et al. [26]	2012	Retrospective review	4	low
Gu et al. [27]	2017	Retrospective study	4	Low
Macedo et al. [28]	2017	Retrospective review	4	Low
Mahmoud [29]	2017	Prospective comparative	3	Moderate
Scaglioni et al. [30]	2018	Retrospective review	4	Moderate
Khan et al. [31]	2018	Retrospective review	4	Low

There was significant heterogenicity in the reported cases. Among 14 studies, the average age and/or range were reported or determined [10,15-17,19,20,22,23,25-27,29-31] but was absent in the remaining four studies [18,21,24,28]. All studies reported on the etiology of the heel defect. The maximum diameter in centimeters of the heel defects was reported in 10 out of the 18 studies, and not reported in eight studies [15,16,20,24,26,28,29,31]. The average follow-up or range of follow-up was reported in 14 studies but not mentioned in four studies [15,16,28,29]. All included studies reported on minor flap complications. Eleven studies reported on the loss of protective sensation of the MPA flap [10,15-18,22,25-28,31] while five studies went on further to report on the comparison of protective sensation of the reconstructed flap to the contralateral normal side [17,22,26,28,31]. Seventeen studies reported on donor site complications and 12 of them reported on the post flap reconstruction ambulatory status of the patient. The largest study had 51 flaps while the smallest had five flaps [10,15].

The total number of medial plantar artery flaps was 277 as shown in Table 2. The two most common etiologies for heel defects were ulcers (n=124 [45.3%]) and trauma (n=98 [35.8%]) followed by tumor, burn, scar, and infection in decreasing frequency. Out of the studies that reported a follow-up period, the follow-up ranged from three months to 44 months. The largest diameter of heel defect reconstructed with the MPA flap was 13 cm.

**Table 2:** Patient and wound characteristics >, Greater than NR, Not reported

Publication	Number of flaps	Indications for flap	Age: mean(range)/years	Largest diameter of wound(cm)	Average Follow up (months)
Reading [15]	5	Trauma x 1, Ulcer x 3, Burn x 1	50.6 (22-78)	NR	NR
Amarante et al. [16]	7	Trauma x3, Ulcer x 4	36.1 (28-47)	NR	NR
Miyamoto [17]	12	Trauma x 5, Ulcer x 1, Tumor x 2, Burn x 4	39.3 (4-79)	13	44
Baker et al. [18]	8	Trauma x 4, Ulcer x2, Burn x1	NR	11	>6
Gravem [19]	24	Ulcer x 24	43.4 (18-75)	6	7
Rashid et al. [20]	20	Trauma x 17, Ulcer x 1, Tumor x 2	28 (22-37)	NR	>3
Benito-Ruiz et al. [21]	6	Trauma x 1, Ulcer x 3, Tumor x 2	NR	5	24
Mourougayan [22]	12	Trauma x 6, Ulcer x 1, Tumor x 5	46 (25-65)	11	9-42
Schwartz [10]	51	Ulcer x 50, Burn x1	50 (6-77)	8	14
Chaudhry et al. [23]	21	Ulcer x 21	50 (6-77)	8	14
Oh et al. [24]	8	NR	NR	NR	>6
Yang et al. [25]	15	Trauma x 7, Ulcer x 3, Tumor x 5	38 (18-58)	12	12
Siddiqi [26]	18	Trauma x 13, Ulcer x 2, Scar x 3	20.2 (6-60)	NR	78
Gu et al. [27]	11	Trauma x 2, Infection x 2, Tumor x 7	38.5 (21-56)	5.5cm	19.6
Macedo et al. [28]	10	Trauma x 10	NR	NR	NR
Mahmoud [29]	14	Trauma x12, Tumor x 1, Ulcer x 1	37.7	NR	NR
Scaglioni [30]	20	Trauma x 2, Ulcer x 8, Tumor x10	54.7 (12-84)	9.5cm	9.25
Khan et al. [31]	16	Trauma x 15, Burn x 1	23 (6-56)	NR	11

As illustrated in Table 3, the total flap survival rate was 98.3% with 272 of 277 flaps surviving. The reported minor flap complications included partial flap necrosis (n=9 [3.2%]), delayed flap healing (n=8 [2.9%]), infection (n=8 [2.9%]) and hematoma (n=1 [0.4%]). Of the studies that reported on the outcomes of protective sensation after flap reconstruction, one of 148 (0.7%) flaps lost protective sensation. Five of the studies compared the protective sensation of the reconstructed flap to the contralateral normal side. Four (4) of the studies concluded that although protective sensation was present, it was inferior compared to the contralateral normal side [17,26,28,31] and one study concluded that the two groups were comparable [22]. For patients that were ambulatory prior to undergoing MPA flap reconstruction, they all remained fully ambulatory after the flap reconstruction. The total rate of reported donor site complication was 5.2% including partial loss of split-thickness skin graft (STSG), hyperkeratosis, delayed healing, and lack of sensation at the instep.

**Table 3:** Summary of outcomes STSG, Spit-thickness skin graft

Publication	Complete flap healing( rate)	Minor flap complication	Loss of protective sensation (comparison to contralateral normal side)	Donor site complications	Ambulatory status after successful flap
Reading [15]	5/5 (100%)	Partial flap necrosis x 3	None	None	Not reported
Amarante et al [16]	7/7 (100%)	None	None	None	Not reported
Miyamoto [17]	11/12(92%)	None	None (inferior to contralateral side)	Partial loss of STSG X3	Fully ambulatory
Baker et al [18]	8/8 (100%)	Abscess x 1, Hematoma x1	None	Hyperkeratosis x 2	Fully ambulatory
Gravem [19]	23/24 (96%)	Partial flap necrosis x 1, Infection x3, Delayed healing x5	Not reported	None	Not reported
Rashid et al [20]	20/20 (100%)	None	Not reported	Partial loss of STSG X2	Fully ambulatory
Benito-Ruiz et al [21]	5/6 (83%)	None	Not reported	None	Fully ambulatory
Mourougayan [22]	12/12 (100)	None	None (comparable to contralateral side)	None	Fully ambulatory
Schwartz [10]	50/51 (98%)	Infection x 3	None	Necrosis of skin bridge x 1	Not reported
Chaudhry et al [23]	20/21(98%)	Infection x3, Delayed wound healing x3	Not reported	None	Not reported
Oh et al [24]	8/8 [100%]	partial flap necrosis x3	Not reported	Not reported	Fully ambulatory
Yang et al [25]	15/15 (100%)	None	None	Lack of sensation at donor site x 3	Fully ambulatory
Siddiqi et al [26]	18/18 (100%)	None	None (inferior to contralateral side)	Partial loss of STSG X1	Fully ambulatory
Gu et al [27]	11/11 (100%)	None	1	None	Fully ambulatory
Macedo et al [28]	10/10 (100%)	Partial flap necrosis x 1	None (inferior to contralateral side)	Partial loss of STSG X1	Not reported
Mahmoud [29]	14/14 (100%)	Partial flap necrosis x 1, infectionx1	Not reported	delayed graft healing x1	Fully ambulatory
Scaglioni [30]	20/20 [100%]	None	Not reported	none	Fully ambulatory
Khan et al [31]	15/16 [ 94%]	None	None (inferior to contralateral side)	None	Fully ambulatory
Total	272/277 [ 98.2%]	Partial flap necrosis:(9/277) [3.2%], Infection: (8/277) [2.9%], Delayed flap healing: (8/277) [2.9%], Hematoma: (1/277) [0.4%], Combined: 26/277 [9.4%]	1/148:[ 0.7%]	14/269: 5.2%	

Assessment of Publication Bias

Publication bias using a funnel plot could not be performed due to the fact that most of the studies were not controlled to allow generation of odds ratios or relative risks. The PRISMA checklist for the study is illustrated in Table 4 (appendix).

Discussion

This systematic review is the first to evaluate the use of the medial plantar artery flap for heel reconstruction. Several studies have documented its safety and viability in foot defect reconstruction. It is more robust than options such as skin graft and local flaps and associated with less morbidity compared to free flaps. This systematic review examines the rate of flap survival and other outcomes after MPA flap reconstruction to the heel. Since its first description in the literature, there have been multiple variations the instep flap including the island pedicled flap and the perforator flap.

The findings of this review are that medial plantar artery flap for heel reconstruction is associated with very high flap survival rate (98.2%), low minor flap complications (9.4%), and low donor site complications (5.2%). However, the included studies were heterogeneous and the outcomes reported were inconsistent. The results from this review are consistent with the largest study on the use of MPA or heel reconstruction by Schwarz [10] with a flap survival rate of 98% compared to 98.2% from this review.

One other flap that has been used for heel reconstruction is the reverse sural artery flap. A few studies have compared this flap to the medial plantar artery flap for heel reconstruction and have found the medial plantar artery flap to have less associated complications [20,29]. Moreover, the donor tissue used in the sural artery flap does not provide the glabrous tissue that the instep provides.

Another advantage of the medial plantar artery flap is the ability to transfer it as a sensate flap. This is very important to protect the foot from injuries including development of heel ulcers. In this review, only 1/148 patients lost protective sensation. Of the few studies that attempted to compare the reconstructed flap to the contralateral normal side, most concluded the presence of protective sensation although was inferior to the contralateral side [17,26,28,31]. This feature of the MPA makes it superior to skin grafts, local flaps, and other non-sensate flaps.

There are several limitations to this review. Firstly, the quality of the included studies was not very high. The highest level of evidence was 3, with GRADE quality level ranging from “very low” to “moderate” for included studies. Secondly, there was significant heterogenicity of data amongst the included studies in terms of study design, reported demographics, and outcomes. Most of the studies were not comparative studies and this, combined with the heterogenicity in reported outcomes made it impossible to perform a meta-analysis. Sub-group analysis could not be performed to determine factors associated with negative outcomes such as diabetes, and other systemic diseases. Whilst we employed a rigorous search strategy, there may be evidence that was not captured during our literature search.

## Conclusions

This systematic review has shown that the medial plantar artery flap is a viable option for reconstruction of heel defects. It has a very high flap survival rate and low rate of minor flap complication as well as donor site complications. The flap provides protective sensation to the reconstructed heel although the protective sensation may be inferior to the contralateral normal side.

## Appendices

**Table 4:** PRISMA checklist

Section/topic	#	Checklist item	Reported on section
TITLE			
Title	1	Identify the report as a systematic review, meta-analysis, or both.	Title
ABSTRACT			
Structured summary	2	Provide a structured summary including, as applicable: background; objectives; data sources; study eligibility criteria, participants, and interventions; study appraisal and synthesis methods; results; limitations; conclusions and implications of key findings; systematic review registration number.	Abstract
INTRODUCTION			
Rationale	3	Describe the rationale for the review in the context of what is already known.	Introduction
Objectives	4	Provide an explicit statement of questions being addressed with reference to participants, interventions, comparisons, outcomes, and study design (PICOS).	Introduction
METHODS			
Protocol and registration	5	Indicate if a review protocol exists, if and where it can be accessed (e.g., Web address), and, if available, provide registration information including registration number.	Research design
Eligibility criteria	6	Specify study characteristics (e.g., PICOS, length of follow-up) and report characteristics (e.g., years considered, language, publication status) used as criteria for eligibility, giving rationale.	Selection criteria
Information sources	7	Describe all information sources (e.g., databases with dates of coverage, contact with study authors to identify additional studies) in the search and date last searched.	Search methodology and strategy
Search	8	Present full electronic search strategy for at least one database, including any limits used, such that it could be repeated.	Search methodology and strategy
Study selection	9	State the process for selecting studies (i.e., screening, eligibility, included in systematic review, and, if applicable, included in the meta-analysis).	Selection criteria
Data collection process	10	Describe method of data extraction from reports (e.g., piloted forms, independently, in duplicate) and any processes for obtaining and confirming data from investigators.	Data extraction and synthesis
Data items	11	List and define all variables for which data were sought (e.g., PICOS, funding sources) and any assumptions and simplifications made.	N/A
Risk of bias in individual studies	12	Describe methods used for assessing risk of bias of individual studies (including specification of whether this was done at the study or outcome level), and how this information is to be used in any data synthesis.	Assessment of study quality and biases
Summary measures	13	State the principal summary measures (e.g., risk ratio, difference in means).	N/A
Synthesis of results	14	Describe the methods of handling data and combining results of studies, if done, including measures of consistency (e.g., I^2^) for each meta-analysis.	Data extraction and synthesis
